# Prevalence of myeloma precursor state monoclonal gammopathy of undetermined significance in 12372 individuals 10–49 years old: a population-based study from the National Health and Nutrition Examination Survey

**DOI:** 10.1038/bcj.2017.97

**Published:** 2017-10-20

**Authors:** O Landgren, B I Graubard, S Kumar, R A Kyle, J A Katzmann, K Murata, R Costello, A Dispenzieri, N Caporaso, S Mailankody, N Korde, M Hultcrantz, T M Therneau, D R Larson, J R Cerhan, S V Rajkumar

**Affiliations:** 1 Myeloma Service, Department of Medicine, Memorial Sloan-Kettering Cancer Center, New York, NY, USA; 2 Divsion of Cancer Epidemiology and Genetics, Biostatistics Branch, National Cancer Institute, Rockville, MD, USA; 3 Division of Hematology, Department of Medicine, College of Medicine, Mayo Clinic, Rochester, MN, USA; 4 Department of Laboratory Medicine and Pathology, College of Medicine, Mayo Clinic, Rochester, MN, USA; 5 Multiple Myeloma Section, Center for Cancer Research, Lymphoid Malignancies Branch, National Cancer Institute, Rockville, MD, USA; 6 Division of Biostatistics, Department of Medicine, College of Medicine, Mayo Clinic, Rochester, MN, USA; 7 Division of Epidemiology, Department of Health Sciences Research, College of Medicine, Mayo Clinic, Rochester, MN, USA

## Abstract

We studied the prevalence of monoclonal gammopathy of undetermined significance (MGUS) in younger individuals, age 10–49 years, using samples from the National Health and Nutritional Examination Survey (NHANES) III. NHANES prevalence rates were standardized to the 2000 US total population. Among 12 372 individuals (4073 blacks, 4146 Mexican-Americans, 3595 whites, and 558 others), MGUS was identified in 63 persons (0.34%, 95% CI 0.23–0.50). The prevalence of MGUS was significantly higher in blacks (0.88%, 95% CI 0.62–1.26) compared with whites (0.22%, 95% CI 0.11–0.45), *P*=0.001. The prevalence of MGUS in Mexican-Americans was at an intermediate level (0.41%, 95% CI 0.23–0.73). The disparity in prevalence of MGUS between blacks and whites was most striking in the 40–49 age-group; 3.26% (95% CI 2.04–5.18) versus 0.53% (95% CI 0.20–1.37), *P*=0.0013. There was a trend to earlier age of onset of MGUS in blacks compared with whites. MGUS was seen in only two persons in the 10–19 age-group (both Mexican-American), and in three persons in the 20–29-year age-group (all of whom were black). In persons less than 50 years of age, MGUS is significantly more prevalent, with up to 10 years earlier age of onset, in blacks compared with whites.

## Introduction

Over 22 000 Americans are diagnosed annually with multiple myeloma.^1^ The estimated US prevalence is rising, and this trend is likely to continue due to improvements in diagnosis and therapy. In 2009, a definitive analysis of a cohort of 77 000 people enrolled in a prospective population-based cancer screening trial showed that multiple myeloma is consistently preceded by a precursor state, monoclonal gammopathy of undetermined significance (MGUS).^2^

There is marked racial disparity in age of onset as well as incidence of multiple myeloma.^3^ Among whites in the US the median age at multiple myeloma diagnosis is 70 years while African-Americans have a median age of 66 years. Importantly, African-Americans have a twofold or greater risk for multiple myeloma compared with whites, and the disparity that is even more pronounced (more than threefold higher risk) in those less than 40 years of age.^4^ The higher incidence of multiple myeloma in blacks results from a higher prevalence of the precursor lesion, MGUS, while the risk of progression from MGUS to multiple myeloma is the same.^5, 6, 7, 8, 9^

Recently, in the first screening study of MGUS with available risk factor data, we were able to study 12 482 adults 50 years or older to accurately quantify the prevalence of MGUS by race and ethnicity in a stratified random sampling of the entire US population.^7^ In that study we found the adjusted prevalence of MGUS to be significantly higher in blacks (3.7%) compared with whites (2.3%). For both groups, the prevalence of MGUS increased with advancing age, but blacks displayed consistently higher prevalence rates at a given age. The prevalence of MGUS in blacks 50–59 years was similar to that of whites 60–69 years, suggesting an earlier age of onset for MGUS in blacks. Indeed, smaller studies have observed an excess of MGUS among blacks younger than 50 years of age; however, to date, no population-based MGUS screening study has been conducted focusing on children and young adults across racial/ethnic groups.

Based on these observations, we hypothesized that the age of onset of MGUS occurs earlier in blacks, and that this can be detected by racial disparities in the incidence of MGUS in persons less than 50 years of age. We utilized samples and data from the National Health and Nutrition Examination Survey (NHANES), a nationally representative sample of the US population, to address this question and to expand our knowledge on patterns of MGUS in individuals younger than 50 years. As a logical extension to our previous NHANES study focusing on 12 482 individuals of age 50 years or older,^7^ we studied the prevalence patterns of MGUS by race and ethnicity in children (10–18) and adults (19–49 years of age) by utilizing serum samples and other variables collected through NHANES.

## Subjects and methods

### Study population and serum samples

The data for this paper come from NHANES III (1988–1994), a large population-based cross-sectional study that includes in-person home interviews and medical examinations conducted by the Centers for Disease Control and Prevention (CDC) to assess the health and nutritional status of adults and children in the United States.^7, 10, 11^ Participant identification is based on a stratified multistage complex sample design to select a nationally representative sample of the civilian, non-institutionalized US population, with oversampling (higher sampling rates) of older adults and non-Hispanic blacks and Mexican-Americans, and other groups. There were 14 129 individuals in the NHANES III sample who were 10–49 years old at home interview who participated in the medical examination component. Of these individuals, 12 372 individuals (88%) had serum samples available for MGUS testing. Because of the loss of observations due to insufficient serum samples used for the MGUS testing, the sample weights provided by NCHS for the medical examination component sample were further adjusted by post-stratification using age (10–11, 12–19, 20–29, 30–39, 40–49) by sex by race/ethnicity (non-Hispanic white, non-Hispanic black, Mexican-American, and other races) categories to make the weighted sample for individuals in NHANES with MGUS evaluation representative of the US population. These categories were similar to those used by NCHS for post-stratifying the original medical examination component weights. Survey data, including demographics, health status, health disorders, behaviors and so on, are collected through household interviews. Physical examinations and blood collection were conducted at a mobile examination center.

### Testing for monoclonal proteins

Testing for the presence of MGUS was performed at the Protein Immunology Laboratory, Mayo Clinic, Rochester, MN, USA, using laboratory techniques that have been well described previously.^12^ Briefly, conventional agarose-gel electrophoresis was performed on sera from all subjects. Samples with an equivocal or definite M-protein present on electrophoresis were then subjected to serum protein immunofixation, and to serum-free light-chain assay for confirmation and typing of the M-protein. All testing and interpretation was done by individuals blinded to all demographic and other details pertaining to the samples being tested.

### Prevalence estimates and risk factors

Once testing was completed, data were transferred to the National Center for Health Statistics for linking to the NHANES Public Use files that contain the serologic and demographic data needed for these analyses. Once the MGUS data were released to the public on the NHANES website, the data could be analyzed by the National Cancer Institute. The prevalence of MGUS was estimated in the total population, as well as for whites, blacks, and Mexican-Americans separately, and by age, gender and other known risk factors.

### Statistical analysis

Prevalence rates were calculated by dividing the sample weighted number of persons with MGUS by the sample weighted number of total persons (with or without MGUS). Logistic regression was used to perform adjusted analyses for the confounders such as age, race and sex.^13^ Wald F-tests, which take account of the complex design of NHANES, were used to test hypotheses regarding the estimated prevalence of MGUS. A restricted cubic spline was used in logistic regression analyses to estimate the relationship of MGUS prevalence on age.^13, 14^ Risk factors for MGUS were studied using available survey information (including questionnaires concerning health behavior, and demographics) available from NHANES. Based on small numbers, we first explored risk factors involving a predefined set of variables that, based on prior studies, have been suggested as having potential associations with MGUS—namely, age, race/ethnicity, obesity, socioeconomic status, and smoking.^7, 12, 15, 16, 17, 18^ We explored the role of socioeconomic status by using poverty index ratio as a proxy marker. All analyses were conducted using SAS version 9.3 (SAS Institute, Cary, NC, USA) and SUDAAN version 11.0.0 (Research Triangle Institute, Research Triangle Park, NC, USA) software programs. All analyses accounted for the sample weighting and complex sample design of the NHANES, as well as correction for nonresponse. All hypothesis tests were determined significant for two-sided *P*-values<0.05 that were not adjusted for multiple comparisons. Because of the small number of individuals with MGUS in the NHANES sample, the *P*-values and confidence intervals will be approximate and should be interpreted with caution.

## Results

### Prevalence

**Figure 1:**
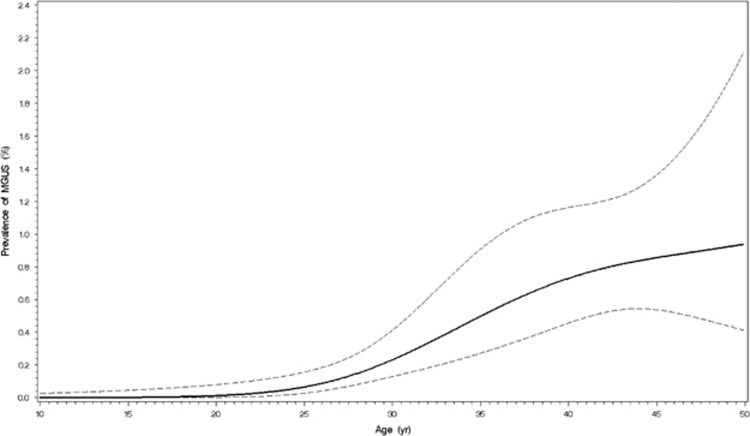
Prevalence of MGUS among 10–49-year olds in NHANES III. The solid line is the estimated prevalence from 3-knot cubic restricted regression spline using logistic regression unadjusted for any covariates. The dash lines show the upper and lower 95% pointwise confidence limits.

Of 14 153 study subjects aged 10 to 49 years of age enrolled in NHANES III or NHANES (1999–2004), stored serum samples to test for monoclonal proteins were available for testing in 12 372 persons (4073 non-Hispanic blacks (considered ‘black’); 4146 Mexican-Americans; 3595 non-Hispanic whites (considered ‘white’); and 558 ‘others’). MGUS confirmed on serum immunofixation was identified in 63 participants, for an overall prevalence of 0.34% (95% confidence interval (CI), 0.23–0.50) (Table 1). MGUS was detected in 31 of 6663 sampled females, as compared with 32 of 5709 sampled males, and prevalence rates were non-significantly higher (*P*=0.41) in males (0.41% 95% CI, 0.22–0.74) than in females (0.28% 95% CI, 0.17–0.46). The unweighted median age among MGUS for both sexes was 42 years. The incidence of MGUS increased with age as shown in Figure 1.

Of the 63 persons in whom MGUS was detected, 34 were black, 9 were white, 17 were Mexican-American, 3 were considered ‘other’. The prevalence of MGUS was significantly higher in blacks (0.88%, 95% CI 0.62–1.26) compared with whites (0.22%, 95% CI 0.11–0.45), *P*=0.001. The prevalence of MGUS in Mexican-Americans was intermediate between that observed in blacks and whites (0.41%, 95% CI 0.23–0.73) and was lower than blacks, *P*=0.023 (Table 1). The age-adjusted prevalences of MGUS for whites, blacks and Mexican-Americans were 0.21 (95% CI 0.11–0.42), 0.99 (95% CI 0.69–1.42), 0.55 (95% CI 0.32–0.95), respectively with a lower prevalence among whites versus blacks, *P*<0.001

When we determined the earliest age of MGUS diagnosis by race/ethnic group, the youngest white, black, and Mexican-American, MGUS cases were 33, 22 and 13 years old, respectively. In the age-group 10–19 (*N*=3590) there were only two MGUS cases (both Mexican-American) and in the age-group 20–29 (*N*=3252) there were three MGUS cases (all of whom were black) (Table 1). There was an observed trend to earlier age of onset of MGUS in blacks compared with whites, and the disparity in prevalence of MGUS between blacks and whites was most striking in the 40–49 age-group; 3.26% (95% CI 2.04–5.18) versus 0.53% (95% CI 0.20–1.37), *P*=0.001 (Table 1).

Because we found evidence of an excess risk of MGUS in the Midwest regions of the US in our previous study focusing on individuals 50 years or older,^7^ in the current study, we examined MGUS prevalence by region of the country in the NHANES III sample because region is not publicly available in NHANES 1999–2004. The unadjusted prevalence combining the North and Midwest regions was 0.33% (95% CI, 0.19–0.59%) versus 0.35% (95% CI, 0.21–0.57%) in the combined South and West regions, respectively. After adjusted for age, sex and race using logistic regression there was no significant difference *P*=0.74 between North and Midwest versus South and West.

### Risk factors

Although the number of MGUS cases overall were small (*N*=63), we explored risk factors based on prior studies including: body mass index,^15, 19, 20, 21^ smoking and socioeconomic status (categorized as high versus low by using poverty index ratio <1 versus⩾1).^15, 16, 17, 18^ Prevalence of MGUS was not statistically associated with body mass index (data not shown). Unadjusted prevalence of MGUS was highest among former smokers, lowest among never smokers (*P*=0.03), and intermediate among former smokers (*P*=0.18) (Table 2). These patterns were similar in each racial group, although African-American current smokers had the highest prevalence of MGUS. After adjustment for age, race, and gender the overall association was no longer statistically significant (*P*=0.11). Lower poverty income-ratio (Table 3) was not significantly associated with the prevalence of MGUS for unadjusted or adjusted, *P*=0.092 and *P*=0.080, respectively.

### Laboratory characteristics of MGUS

The laboratory characteristics of the MGUS detected in this study are shown in Table 4; these factors have a direct impact on the risk and type of progression. IgA subtype appeared more frequent in whites. The unweighted median monoclonal protein concentration was similar (0.1 gm/dl) in blacks, whites and Mexican-Americans. However, the range of individual M-protein values was observed to be wider in whites (0.1–2.4 g/dl) compared with blacks (0.1–1.5 g/dl) (Table 4).

## Discussion

Multiple myeloma is significantly more common in blacks,^3^ which in turn is due to a higher prevalence of myeloma precursor disease (MGUS) in blacks.^5^ Our previous study using a systematic population-based sample from NHANES of individuals 50 years of age and older showed significantly higher prevalence of MGUS in blacks compared with whites in all age-strata.^7^ Based on those observations, we hypothesized that MGUS starts at an earlier age in blacks, and that a racial disparity in the prevalence of MGUS would be apparent even before age 50. This was further supported by an earlier age of onset of multiple myeloma in blacks.^4^ The present study represents the first and largest population-based screening study of MGUS with available risk-factor data in individuals younger than 50 years of age. We studied 12 372 persons ages 10–49 enrolled in NHANES III on whom samples were available for testing, representing 88% of the total persons enrolled in the medical examination component sample. We are therefore able to quantify the prevalence of MGUS by race and ethnicity in a sample of the US under the age of 50 years.

We show that the prevalence of MGUS is indeed significantly higher in blacks compared with whites in the younger age group studied. This study also finds that the prevalence of MGUS in Mexican-Americans 10–49 years of age is in-between those of blacks and whites. The study suggests that MGUS may start at an earlier age in blacks compared with whites. In the 20–29 age-group, MGUS was not identified in whites, but was seen in 0.26% of blacks. Among persons 30–39 years of age, prevalence of MGUS was 2.5 times higher in blacks compared with whites, while in the 40–49 age-group, the prevalence of MGUS was over sixfold higher in blacks compared with whites, a difference that was statistically significant. Across these two age-groups, the prevalence of MGUS in blacks was similar to that seen in whites a decade (or more) older. These results suggest racial heterogeneity early in the carcinogenic disease pathway, while also consistent with the younger age distribution seen among blacks compared with whites in multiple myeloma. As with our previous study including individuals 50 years or older,^7^ in the current study the prevalence of MGUS increases with age in whites, blacks and Mexican-Americans (Figure 1). We also found that MGUS is rare prior to the age of 30; only five persons below 30 had MGUS.

Uniquely, we were also able to analyze data from the NHANES survey research program to investigate the association of MGUS with several potential risk factors. Our previous studies suggest that racial disparities in MGUS may be related more to genetic rather than environmental differences. We previously found that the increased prevalence of MGUS in African-Americans is also seen in blacks from Ghana, Africa.^6^ Among women of similar socioeconomic status, the prevalence of MGUS was twice as high in African-Americans compared with whites.^15^ These earlier studies had led us to conclude that the disparity in prevalence of MGUS by race was driven mainly by genetic factors. Although it is still possible that environmental factors play a role, evidence to support this is still lacking. In this study, we found an association between smoking and MGUS in all races, but this was not present after adjustment for age, race and gender.

In summary, in this comprehensive screening study, we provide data on the prevalence of MGUS in younger individuals. We also provide the basis for the increased risk of multiple myeloma in blacks, by demonstrating an increase in the prevalence of the precursor lesion MGUS in blacks compared with whites that is apparent even before the age of 50. We also provide data that suggest that MGUS may start at an younger age group in blacks than previously recognized. As in previous studies in older individuals, advancing age and male gender were associated with an increased risk of MGUS. Our study design utilizing the NHANES cohort allows our results and conclusions to be wholly representative of the US population. Our findings have implications for prognosis, counseling, public health policy and future research. Clinically, it is important to be aware that multiple myeloma and MGUS have an earlier age of onset in African-Americans, and physicians who encounter younger individuals with typical symptoms should consider multiple myeloma as a differential diagnosis. Scientifically, it opens up new research avenues designed to uncover underlying biological mechanisms of the early patterns of transformation from MGUS to multiple myeloma.

## Figures and Tables

**Table 1:** Prevalence of monoclonal gammopathy of undetermined significance (MGUS) (%), by race, age and gender

*Variable (# with MGUS)*	*Number of persons with MGUS*	*Blacks (34)*^a^ *% (95% CI)*	*Whites (9)*^a^ *% (95% CI)*	*Mexican-American (17)*^a^ *% (95% CI)*	*Total*^b^ *% (95% CI)*
*Age group, years*					
10–19	2	0.00 (NA)	0.00 (NA)	0.11 (0.02–0.84)	0.01 (0.00–0.07)
20–29	3	0.26 (0.08–0.84)	0.00 (NA)	0.00 (NA)	0.03 (0.01–0.11)
30–39	17	0.81 (0.43–1.53)	0.32 (0.11–0.99)	0.23 (0.06–0.79)	0.49 (0.27–0.91)
40–49	41	3.26 (2.04–5.18)	0.53 (0.20–1.37)	2.20 (1.22–3.95)	0.88 (0.53–1.47)
					
*Sex*					
Male	32	0.84 (0.54–1.29)	0.26 (0.10–0.68)	0.47 (0.25–0.87)	0.41 (0.22–0.74)
Female	31	0.92 (0.58–1.48)	0.18 (0.06–0.53)	0.36 (0.16–0.78)	0.28 (0.17–0.46)
					
Total	63	0.88 (0.62–1.26)	0.22 (0.11–0.45)	0.41 (0.23–0.73)	0.34 (0.23–0.50)

Abbreviation: CI, confidence interval; NA, not applicable.

a Number in parenthesis here indicates number of persons with MGUS in that race/ethnicity group.

b Includes ‘Other’ race/ethnicity group.

**Table 2:** Prevalence of MGUS by smoking status

*Race*	*No. of persons with MGUS*^a^	*Smoking status*	*Prevalence of MGUS (%)*	*Lower limit of 95% CI*	*Upper limit of 95% CI*
All persons	20	Never	0.22	0.11	0.43
	14	Former	0.76	0.33	1.76
	28	Current	0.51	0.24	1.13
Whites	3	Never	0.18	0.056	0.58
	5	Former	0.75	0.27	2.07
	1	Current	0.08	0.01	0.62
Blacks	11	Never	0.53	0.29	0.96
	3	Former	1.10	0.33	3.61
	20	Current	2.16	1.38	3.37
Mexican-American	5	Never	0.23	0.08	0.67
	6	Former	1.24	0.51	3.00
	5	Current	0.76	0.30	1.92

Abbreviations: CI, confidence interval; MGUS, monoclonal gammopathy of undetermined significance.

a Because of missing smoking data there are 62 persons with MGUS in this table.

**Table 3:** Prevalence of MGUS by poverty status

*Race*	*No. of persons with MGUS*^a^	*Poverty status*	*Prevalence of MGUS (%)*	*Lower limit of 95% CI*	*Upper limit of 95% CI*
Total	16	Below poverty	0.72	0.28	1.87
Total	42	Not below poverty	0.26	0.17	0.41
Whites	1	Below poverty	0.46	0.06	3.27
Whites	7	Not below poverty	0.18	0.08	0.40
Blacks	9	Below poverty	0.85	0.42	1.71
Blacks	23	Not below poverty	0.95	0.59	1.53
Mexican-American	5	Below poverty	0.32	0.12	0.82
Mexican-American	11	Not below poverty	0.51	0.30	0.86

Abbreviations: CI, confidence interval; MGUS, monoclonal gammopathy of undetermined significance.

a Because of missing data there are 58 persons with MGUS in this table.

**Table 4:** Characteristics of the subjects with monoclonal gammopathy of undetermined significance (MGUS), by race/ethnicity

*Total,* n	*Black (*N=*34)*	*White (*N=*9)*	*Mexican-American (*N=*17)*
Male sex % (95% CI)	44 (30.6–58.2)	59.6 (25.8–86.2)	58.9 (39.6–75.7)
			
*Age, categories, % (95% CI)*			
10–19	0	0	8.2 (1.2–38.9)
20–29	7.7 (2.4–22.4)	0	0
30–39	24.3 (12.8–41.5)	42.6 (4) (14.8–76.0)	12.7 (3.5–36.8)
40–49	67.9 (50.3–81.6)	57.4 (24.0–85.2)	79.2 (54.2–92.4)
			
*Immunoglobulin isotype, % (95% CI)*			
IgG	74.0 (53.0–87.8)	72.9 (33.9–93.4)	60.9 (28.4–85.9)
IgA	10.0 (2.7–30.7)	27.1 (6.6–66.1)	18.1 (3.8–55.2)
IgM	0	0	5.3 (0.7–31.5)
Biclonal	16.0 (7.0–32.7)	0	15.7 (3.7–47.8)
			
*Light chain type, % (95% CI)*			
Kappa	45.9 (30.3–62.4)	68.3 (27.8–92.4)	68.6 (47.4–84.2)
Lambda	54.1 (37.6–69.7)	11.1 (1.4–52.3)	31.4 (15.8–52.6)
Biclonal	0	20.5 (2.9–69.2)	0
			
*Monoclonal protein, g/dl*			
Median^a^	0.1	0.1	0.1
Range	0.1–1.5	0.1–2.4	0.1–1.8

Abbreviation: CI, confidence interval.

a Medians are unweighted (that is, not weighted by the sample weights).
